# Draft Genome Sequence of *Mesorhizobium* sp. UFLA 01-765, a Multitolerant, Efficient Symbiont and Plant Growth-Promoting Strain Isolated from Zn-Mining Soil Using *Leucaena leucocephala* as a Trap Plant

**DOI:** 10.1128/genomeA.00050-16

**Published:** 2016-03-10

**Authors:** Wesley Melo Rangel, Sofie Thijs, Fatima Maria de Souza Moreira, Nele Weyens, Jaco Vangronsveld, Jonathan D. Van Hamme, Eric M. Bottos, Francois Rineau

**Affiliations:** a Biology Department, Federal University of Lavras (UFLA), Lavras, Minas Gerais, Brazil; b Soil Science Department, UFLA, Lavras, Minas Gerais, Brazil; c Centre for Environmental Sciences, Hasselt University, Diepenbeek, Belgium; d Department of Biological Sciences, Thompson Rivers University, Kamloops, Canada

## Abstract

We report the 7.4-Mb draft genome sequence of *Mesorhizobium* sp. strain UFLA 01-765, a Gram-negative bacterium of the *Phyllobacteriaceae* isolated from Zn-mining soil in Minas Gerais, Brazil. This strain promotes plant growth, efficiently fixes N_2_ in symbiosis with *Leucaena leucocephala* on multicontaminated soil, and has potential for application in bioremediation of marginal lands.

## GENOME ANNOUNCEMENT

Native rhizobia from metal-contaminated mine sites are promising candidates for use in reclamation, particularly in phytoremediation.

We isolated an efficient N_2_-fixing and plant growth-promoting bacterium from Zn-contaminated mining soil in Minas Gerais, Brazil. Identified as a *Mesorhizobium* sp. by partial 16S rRNA gene sequencing (99% identity to *Mesorhizobium* sp. strain CCANP87, Genbank accession no. HF931067) (1), strain UFLA 01-765 was selected for genome sequencing.

Genomic DNA was isolated from stationary-phase cells using a DNeasy blood and tissue kit (Qiagen, Venlo, the Netherlands), treated with RNase I and purified by phenol:chloroform extraction. A library was constructed according to Thijs et al. (2) prior to sequencing on an Ion Torrent PGM (Life Technologies Inc., Carlsbad, CA).

In total, 1.2 million reads (mean length 291 bases) generated 351 Mb of data (>305 M Q20 bases) in Torrent Suite 4.2.1. These were assembled using SPAdes 3.1.0 (3, 4) (uniform coverage mode; kmers 21, 33, 55, 77, and 99) into 185 contigs greater than 500 bp, giving a consensus length of 7,464,539 bp (largest contig 366,840 bp; *N*_50_ = 123,481 bp). Contigs were ordered with the closest related reference genome, that of *Mesorhizobium loti* MAFF303099 (accession no. BA000012) using Mauve (5). UFLA 01-765 has a GC content of 56.17%, 49 tRNAs, 3 rRNAs (5S, 16S, and 23S), and 7,084 genes. A total of 5,899 proteins were assigned to clusters of orthologous group (COG) families through the NCBI Prokaryotic Genome Annotation Pipeline (PGAP) (6). Functional annotation was carried out using the RAST (7) server, generating 409 subsystems.

In symbiosis with *Leucaena leucocephala* growing on Cd-, Pb-, and Zn-contaminated soil, UFLA 01-765 promotes plant growth, increases nitrogen accumulation, and decreases glutathione reductase (EC 1.8.1.7) and guaiacol peroxidase (EC 1.11.1.7) activities in tissues of its host plant growing on metal-contaminated soil. Genome analysis confirmed the presence of genes coding for multiresistance, including metal-dependent hydrolases of the beta-lactamase superfamily I (similar to that of *Mesorhizobium* sp. BNC1 and *Sinorhizobium meliloti* 1021), type I secretion outer membrane proteins, a DNA-binding metal response regulator (similar to *S. meliloti* 1021), and the cobalt-zinc-cadmium resistance protein CzcD. Several genes involved in plant growth promotion (auxin biosynthesis, 1-aminocyclopropane-1-carboxylate deaminase activity, siderophore production, phosphorus solubilization, and N_2_ fixation) were found and their activity was confirmed in phenotypic tests. Strain UFLA 01-765 produces indole-3-acetic acid (IAA), 1-aminocyclopropane-1-carboxylate (ACC) deaminase, and siderophores, solubilizes Ca_3_(PO_4_)_2_, and utilizes glycerol, glucose, fructose, and sucrose as carbon sources.

*Bradyrhizobium japonicum* USDA110, *S. meliloti* 1021, and the photosynthetic *Bradyrhizobium* sp. ORS278 can grow autotrophically, and all carry the RuBisCO gene (8, 9). Strain UFLA 01-765 is the first reported *Mesorhizobium* sp. with genes for the Calvin Benson Bassham cycle, ribulose-1,5-bisphosphate carboxylase/oxygenase (RuBisCO), and phosphoribulokinase. Further studies are needed to verify if it can grow chemoautotrophically.

*Mesorhizobium* sp. UFLA 01-765 is a promising inoculant for *L. leucocephala* to stimulate revegetation of Zn- and Cd-contaminated sites, and it is a candidate as a type strain for the genus in studies of chemoautotrophic growth in rhizobia.

### Nucleotide sequence accession numbers.

This whole-genome shotgun project has been deposited at DDBJ/EMBL/GenBank under the accession no. LPWA00000000. The version described in this paper is version LPWA01000000.
